# Circulating dendritic cell precursors in chronic kidney disease: a cross-sectional study

**DOI:** 10.1186/1471-2369-14-274

**Published:** 2013-12-10

**Authors:** Katharina Paul, Daniel Kretzschmar, Atilla Yilmaz, Barbara Bärthlein, Stephanie Titze, Gunter Wolf, Martin Busch

**Affiliations:** 1 Department of Internal Medicine III, Division of Nephrology, Jena University Hospital Friedrich-Schiller University, Erlanger Allee 101, Jena, 07740 Germany; 2 Department of Internal Medicine I, Division of Cardiology and Intensive Care Medicine, Jena University Hospital Friedrich-Schiller University, Erlanger Allee 101, Jena 07740, Germany; 3 Chair of Medical Informatics, University of Erlangen-Nürnberg, Krankenhausstr. 12, Erlangen 91054, Germany; 4 Department of Nephrology and Hypertension, University of Erlangen-Nürnberg, Ulmenweg 18, Erlangen 91054, Germany

## Abstract

**Background:**

Dendritic cells (DC) are professional antigen-presenting cells in the immune system. They patrol the blood as circulating dendritic cell precursors (DCP). Decreased blood DCP count has been shown to be related to atherosclerotic plaque burden. Since chronic kidney disease (CKD) is associated with chronic inflammation and increased cardiovascular risk, the aim of our study was to investigate a potential effect of CKD on circulating DCP numbers especially in patients with a history of cardiovascular disease.

**Methods:**

The number of circulating myeloid (mDCP), plasmacytoid (pDCP), and total DCP (tDCP) was analysed by flow cytometry in 245 patients with CKD stage 3 (with and without known cardiovascular events) and 85 coronary healthy controls. In addition, data were compared with a historical group of 130 patients with known coronary artery disease (CAD).

**Results:**

Compared to controls, patients with CKD 3 revealed a significant decrease in circulating mDCP (-29%), pDCP (-43%), and tDCP (-38%) (*P* < 0.001, respectively). Compared with CAD-patients, the decrease in circulating DCP in CKD was comparable or even more pronounced indicating a potential role for DCP in cardiovascular risk potentiation due to CKD.

**Conclusions:**

Based on previous findings in CAD, the marked decrease of DCP in CKD implicates a potential role for DCP as a mediator of cardiovascular disease. Whether DCP in CKD may act as new cardiovascular biomarkers needs to be established in future prospective trials.

## Background

Dendritic cells (DC) which are derived from bone marrow progenitor cells, are potent antigen-presenting cells. They play a major role in the initiation and maintenance of innate and adaptive immunity and are present in nearly all body tissues forming a dense network [1-3]. These cells can be found in an immature state in the blood as DC precursors (DCP). Their main function is to observe the internal environment to detect foreign potential harmful antigens [4]. In consequence of antigen uptake and by presence of inflammatory stimuli, immature DC undergo terminal differentiation. By this, DC express different costimulatory molecules and thus stimulate the immunogenic system [2,5,6].

In humans, there are at least two different DC-subpopulations: myeloid DC (mDC) and plasmacytoid DC (pDC). The mDC are most similar to monocytes. They consist of two subsets: the more common mDC-1, which is a major stimulator of T-cells and the extremely rare mDC-2, which may have a function in fighting wound infection. The pDC look like plasma cells and are often referred to as interferon-producing cells, but have certain characteristics similar to mDC [7].

The amount of circulating DCP is related to immune status as significant alterations in blood DCP subsets have been demonstrated in a variety of clinical conditions. Due to inflammatory and immunological processes in atherosclerotic plaques, there are close relationships between atherosclerosis, inflammation, and the (auto) immune reactions [3,8].

Previous studies have shown significant changes of the frequency of circulating DCP in peripheral blood during inflammation or myocardial infarction [7,9]. Yilmaz *et al.*[10] showed a decrease in circulating mDC precursors (mDCP) in patients with different manifestations of coronary artery disease (CAD) such as stable and unstable angina pectoris as well as acute myocardial infarction. Based on this observation, recruitment and consumption of DC in atherosclerotic plaques was discussed as a reason for their decrease in the circulation. According to this finding, a significant decrease in circulating levels of mDCP, pDC precursors (pDCP) and total DC precursors (tDCP) was described in a large cohort of patients having stable CAD [11].

Chronic Kidney Disease (CKD) is associated with a high prevalence of distinct cardiovascular (CV) risk factors [12,13]. Moreover, CKD itself is an important CV risk factor. An estimated glomerular filtration rate (eGFR) less than 60 mL/min/1.73 m^2^ is an independent predictor of all-cause and CV mortality [14]. Uremic toxins have proinflammatory effects and their accumulation represents a chronic stimulus to inflammatory response. Thus, elevated levels of inflammatory mediators and an increase in oxidative stress can be observed in CKD [14]. Vascular changes are initiated and perpetuated by the interaction of immune cells with cells of the vessel wall [12,13,15]. Atherosclerosis, vascular calcification, or development of left ventricular hypertrophy and congestive heart failure are the main causes of CV mortality in CKD [16]. Most of the patients with CKD die from CV events (CVE), often before reaching end-stage renal disease (ESRD) [13,16,17]. Patients with CKD stage 3 have a 2 to 4 times higher risk of CV mortality as compared to patients without CKD depending on the stage of CKD, the underlying kidney disease and the extent of albuminuria [18]. In patients with ESRD, cardiovascular mortality is responsible for up to 45% of total mortality [14,19]. On that account, the CV health status of patients with earlier stages of CKD is of particular importance for the prevention of CVE [13].

Disturbed immunological status may contribute to proatherosclerotic mechanisms (i.e. by a decrease of specialized antibodies against plaque antigens or during elimination of oxidized LDL) [19]. Furthermore in CKD, the chronic inflammatory state may lead to a reduction of circulating DCP by consumption in the atherosclerotic vessel wall. Thus, circulating DCP may act as a new biomarker for latent inflammatory processes, probably due to vascular disease and may furthermore reflect plaque burden.

There are less profound data regarding the number of circulating DCP in patients with CKD stage 3. Hesselink *et al*. [5] detected a decrease in circulating DCP in patients with CKD stage 5 compared to healthy volunteers.

The aim of this investigation was to analyse the level of circulating DCP in patients with CKD stage 3 with and without a history of CVE. Findings were compared with DCP counts of coronary healthy controls and patients with known CAD in order to determine coherencies of CV status, DCP count and kidney function.

## Methods

### Participants

Patients with CKD stage 3 with and without a history of CVE were enrolled within the German Chronic Kidney Disease Study (GCKD), which is a German national cohort study [20]. Based on the GCKD protocol [20], 245 patients with CKD stage 3 were included in our cross-sectional study. All patients were recruited in the GCKD regional center of Jena between January and August 2011. In brief, for GCKD, patients are screened to fulfill the criteria of an eGFR between 30-60 mL/min calculated by the 4-variable MDRD formula. Main criteria for exclusion from the GCKD study are described elsewhere [20]. The underlying kidney diseases of this substudy cohort were as follows: 33% hypertensive nephropathy, 17% diabetic nephropathy, 11% glomerulonephritis, 10% interstitial nephritis, 4% polycystic kidney disease and 1% vascular nephropathy, 24% miscellaneaous.

CKD 3 patients who already had myocardial infarction, stroke, peripheral artery disease, or patients who underwent coronary revascularization by intracoronary stenting/ balloon dilatation or coronary artery bypass grafting were defined as patients with a history of CVE. The remaining CKD 3 patients were defined as CKD 3 patients without history of CVE.

Eighty-five otherwise healthy individuals served as coronary healthy controls. They had angina-like symptoms and therefore underwent coronary angiography which led to the exclusion of CAD in all cases. Relevant CKD (by an eGFR <60 mL/min) was excluded in the coronary healthy control group as well as in the CAD group.

Furthermore, data were compared with a well-described cohort of patients having stable CAD, described in 2009 by Yilmaz *et al*. [11]. In these patients, based on the results of coronary angiography, a CAD score was calculated as follows: early CAD (score 1 - 5; n = 43), moderate CAD (score 6 - 10; n = 42) and advanced CAD (score > 10; n = 45) [4].

Moreover, for the present investigation, the intake of any immunosuppressive drugs or suffering from diseases that could interfere with our analysis e.g. any kind of infections, malignancies, autoimmune diseases, and hyperthyreoidism were excluded in patients and controls.

### Description of procedures or investigations undertaken

Blood samples from controls and patients were collected in 9 mL EDTA tubes and immediately cooled (4 -10°C). Samples were analysed by flow cytometry for circulating DCP within 8 h after the collection. The survival and stability of DCP was tested before. Samples were analysed for DCP immediately, after 8 hours and after 24 hours. By this, a time interval of 8 hours was found to be safe (data not shown). For routine blood analyses, especially leukocyte count, the same blood sample as for DCP analysis was used. Routine blood analyses were performed by standardised techniques in the central laboratories of the university hospitals of Jena and Erlangen and in the laboratories of Synlab® Labordienstleistungen (core data set of GCKD). For conventional CRP measurement the lower detection limit was 2 mg/L.

Using the Blood Dendritic Cell Enumeration kit™ (BDCA kit; Miltenyi Biotec) circulating mDCP and pDCP were analysed by four-colour staining and FACS analysis in fresh blood samples collected in tubes containing EDTA. Circulating mDCP and pDCP were identified according to their expression of BDCA-1, BDCA-2 and the absence of the expression of other peripheral blood mononuclear cell (PBMC) markers. Thus the cells were classified according to CD 303, CD1c, CD14, CD19 and CD141. For this purpose, 300 μL of blood were mixed with 20 μL of the control cocktail for isotype control and 300 μL of blood were mixed with 20 μL of the anti-BDCA cocktail for cell staining. In order to discriminate dead cells, 10 μL of a fluorescent cell-impermeant dye (which binds to nucleic acids of dead cells) were added and samples were incubated for 10 min under 60 W light bulb. After cell staining, erythrocytes were lysed using the red blood cell lysis solution from the Blood Dendritic Cell Enumeration kit™ (BDCA kit; Miltenyi Biotec). Cells are then washed and fixed using fix solution. Another solution was added to the samples for optimal dead cell discrimination even after prolonged storage (Miltenyi Biotec).

**Figure 1:**
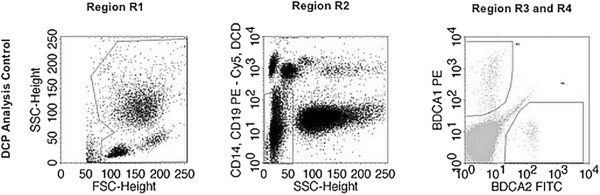
**Gating strategy used for the identification of circulating mDCP, pDCP, and tDCP by flow cytometry.** Region R1 white blood cells (WBC) were separated from debris and platelets using their forward and side scatter (FSC and SSC). Region R2 was used to exclude granulocytes by SSC, B lymphocytes by CD19 staining, monocytes by CD14 staining, and dead cells by propidium iodide-staining. In regions R3 and R4, circulating mDCP and pDCP were detected according to their specific BDCA-1 and BDCA-2 staining, respectively.

Finally, cells were analysed using FACSCalibur flow cytometer with CellQuest software (Becton Dickinson). As circulating DCP comprise only 0.1-1% of white blood cells (WBC), a special gating strategy to exactly analyse the number of mDCP, pDCP, and tDCP was used. In Region R1 2*10^5^ white blood cells (WBC) were registered defined by forward scatter (FSC) and side scatter (SSC). In Region R2, granulocytes were excluded according to their high SSC and lymphocytes, monocytes, and dead cells were excluded according to their CD19, CD14, and propidium iodide staining. Circulating mDCP and pDCP were detected due to their specific staining for BDCA-1 and BDCA-2 in regions R3 and R4 and total DCP (tDCP) were the sum of cells in region R3 and R4 (Figure 1). Thus, the relative cell numbers of circulating DCP were determined as percentage of WBC. Subsequently, the absolute cell numbers (cells/μL) were assessed by multiplying the relative cell numbers with the individual WBC count, which was measured in routine laboratory in the same blood sample. The current investigations as well as the historical CAD cohort were measured by the use of the same laboratory methods including the same type of flow cytometer (FACSCalibur).

For measurement standardisation, a daily and monthly calibration of the flow cytometer was done. Moreover for reliable enumeration of DCP, an isotype control sample was always included for compensation of variations between the measurements.

### Ethics

The study was carried out in accordance with the Declaration of Helsinki (2000) and was approved by the institutional ethics committees from the University of Jena and Erlangen. Written informed consent was obtained from all participants.

### Statistical methods

All values are expressed as medians (minimum, maximum) and means (± standard deviation) or percentages, as appropriate. A *P*-value of < 0.05 was considered to state statistical significance. Student’s t-test (for normal distribution) and Mann-Whitney Rank Sum test (for not-normally distributed data) were used for the comparison between two independent groups. There was no logarithmic (or other) transformation as the data was tested for normal distribution. Categorical clinical data (e.g. gender, presence of hypertension, smoking, diabetic comorbidity) were compared using chi-square statistics. Kruskal-Wallis Test or chi-square statistics were performed for comparison of all 3 groups. Correlation analyses were performed using Pearson Product Moment Correlation for normally distributed data or Spearman Rank Order Test for not-normally distributed data. Controls, CKD 3 and CAD patients differ in several factors (e.g. age, gender), which may influence DCP counts. Therefore a linear regression was performed using DCP count as dependent variable (Additional file 1). Age, male gender, diabetes mellitus, hypertension, smoking, CRP, leukocyte count, GFR, creatinine, cholesterol, HDL, LDL and TG and group dependence were analysed as independent variables. For group dependency, CKD 3 patients were used as the reference group. Data analysis was performed using SigmaStat 3.0® software, SPSS (SPSS Inc., USA), version 19 and Prism 4.0 (GraphPad Software, Inc.).

## Results

### Study population

Clinical data of 245 patients with CKD stage 3 out of the GCKD study cohort [20], 130 patients with stable CAD and 85 coronary healthy controls are shown in Table 1. There was no significant correlation between gender and circulating DCP in the control group (data not shown), whereas a weak correlation of mDCP (r = -0.21, *P* = 0.05) but not pDCP with age was found.

**Table 1:** Clinical data of patients with CKD 3, CAD and control group

	**Control**	**CKD 3**	**P-Value**^**1**^	**CAD**	**P-Value**^**2**^	**P-Value**^**3**^
	**(n = 85)**	**(n = 245)**		**(n = 130)**		
Age (years)	58 (21, 78)	66 (30, 75)	< 0.00	66 (38, 85)	n.s.	< 0.001
	58 ± 11	64 ± 9		64 ± 11		
Male gender (%)	43	60	0.010	57	n.s.	0.035
Diabetes mellitus (%)	13	38	< 0.001	15	< 0.001	< 0.001
Hypertension (%)	71	90	< 0.001	72	< 0.001	< 0.001
Smoking (%) (current and ex-smoking)	38	51	n.s.	43	n.s.	n.s.
C-Reactive protein (mg/L)	2.0 (2.0, 9.0)	2.1 (2.0, 9.4)	n.s.	2.0 (2.0, 9.0)	n.s.	n.s.
	2.9 ± 1.5	3.3 ± 1.9		3.3 ± 1.9		
Leukocytes (Gpt/L)	6.9 (4.2, 12.0)	6.4 (3.6, 12.7)	0.004	7.3 (3.7, 17.1)	< 0.001	< 0.001
	7.1 ± 1.5	6.5 ± 1.6		7.5 ± 2.3		
GFR (mL/min/1.73 m^2^)	83 (60, 215)	46 (30, 60)	< 0.001	72 (60, 119)	< 0.001	< 0.001
	85 ± 20	45 ± 9		75 ± 11		
Creatinine (mg/dL)	0.88 (0.34, 1.26)	1.46 (0.75, 3.35)	< 0.001	0.98 (0.69, 1.30)	< 0.001	< 0.001
	0.87 ± 0.15	1.52 ± 0.46		1.00 ± 0.14		
Cholesterol (mg/dL)	217 (121, 328)	203 (91, 411)	0.001	198 (108, 355)	n.s.	0.008
	222 ± 47	206 ± 49		201 ± 46		
HDL (mg/dL)	56 (24, 108)	48 (21, 119)	0.014	47 (31, 82)	n.s.	0.012
	56 ± 17	51 ± 17		49 ± 11		
LDL (mg/dL)	135 (65, 218)	109 (21, 278)	< 0.001	131 (64, 226)	< 0.001	< 0.001
	141 ± 37	113 ± 43		132 ± 35		
TG (mg/dL)	127 (48, 514)	184 (37, 806)	< 0.001	147 (44, 756)	0.018	< 0.001
	148 ± 80	203 ± 117		176 ± 108		
**DCP count**						
mDCP rel.	0.22 (0.09, 0.58)	0.17 (0.04, 0.44)	< 0.001	0.21 (0.01, 0.47)	<0.001	< 0.001
(% of WBC)	0.24 ± 0.10	0.18 ± 0.07		0.22 ± 0.10		
pDCP rel.	0.13 (0.04, 0.30)	0.08 (0.02, 0.40)	< 0.001	0.11 (0.00, 0.32)	< 0.001	< 0.001
(% of WBC)	0.14 ± 0.06	0.09 ± 0.04		0.11 ± 0.06		
tDCP rel.	0.38 (0.15, 0.76)	0.26 (0.09, 0.58)	< 0.001	0.34 (0.02, 0.72)	< 0.001	< 0.001
(% of WBC)	0.39 ± 0.13	0.27 ± 0.10		0.34 ± 0.14		
mDCP abs.	15.3 (5.2, 34.4)	10.8 (3.2, 35.6)	< 0.001	14.8 (1.5, 35.0)	<0.0001	< 0.001
(cells per μL)	16.8 ± 7.2	11.4 ± 4.7		15.8 ± 7.0		
pDCP abs.	8.9 (2.5, 16.5)	5.0 (1.1, 16.7)	< 0.001	7.4 (0.0, 25.8)	< 0.001	< 0.001
(cells per μL)	9.2 ± 3.7	5.5 ± 2.7		8.4 ± 5.2		
tDCP abs.	27.3 (9.6, 67.8)	16.8 (4.3, 46.2)	< 0.001	24.1 (1.7, 48.8)	< 0.001	< 0.001
(cells per μL)	27.2 ± 10.0	17.5 ± 6.1		25.1 ± 10.5		

^1^CKD 3 compared to control.

^2^CAD compared to CKD 3.

According to Student’s t-test, Mann-Whitney-test or chi-square statistics, as appropriate.

^3^For differences between any group, according to Kruskal-Wallis-Test or chi-square statistics as appropriate.

No significant differences between controls and CKD 3 patients were observed concerning smoking status and C-reactive protein (CRP). Compared to coronary healthy controls, patients with CKD stage 3 were different concerning age, diabetes mellitus, hypertension, LDL, TG (*P* < 0.001, respectively), male gender (*P* = 0.010), leukocyte count (*P* = 0.004), values of total cholesterol (*P* = 0.010) and HDL (*P* = 0.014, Table 1).

Compared to CAD patients, CKD 3 patients were not different concerning age, male gender, smoking, CRP, values of total cholesterol and HDL. CKD 3 patients suffered more often from diabetes mellitus and hypertension (*P* < 0.001, respectively), and had higher values of TG (*P* = 0.018). Instead of this, CAD patients had higher values of leukocytes, and LDL cholesterol (*P* < 0.001, respectively, Table 1).

In the CAD-subgroups according to their different CAD scores, there was no significant difference or trend concerning GFR.

### Circulating DCP in patients with CKD stage 3 vs. controls

Numbers of circulating mDCP, pDCP, and tDCP of controls, patients with CKD stage 3 and CAD patients are given in Table 1.

**Figure 2:**
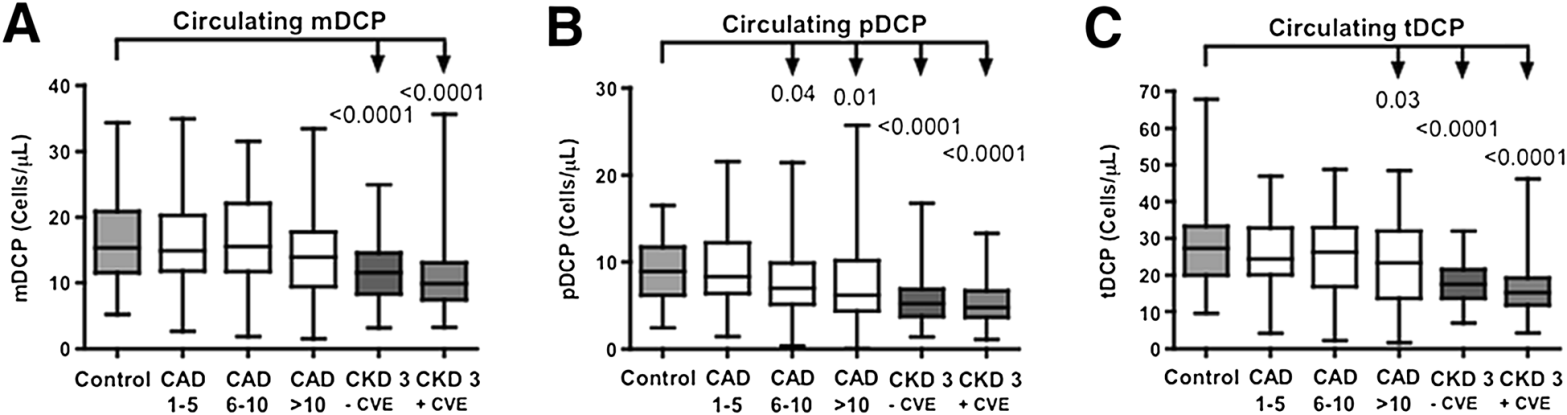
**Absolute numbers of circulating mDCP, pDCP, and tDCP in controls, CAD and CKD 3 patients.** Absolute numbers of circulating mDCP **(A)**, pDCP **(B)**, and tDCP **(C)** (cells/μL) in 85 coronary healthy controls, 130 CAD patients, divided into 3 CAD Scores 1-5 (n = 43), 6-10 (n = 42), >10 (n = 45), and CKD 3 patients without (n = 182) and with (n = 63) CVE (Mann-Whitney-test). Box plots indicate the median (line inside the box), 25^th^ and 75^th^ percentile (lower and upper boundary of the box), and 10^th^ and 90^th^ percentile (whiskers outside the box).

In patients with CKD stage 3, significant lower absolute (Figure 2) and relative numbers (*P* < 0.001, respectively) of circulating mDCP, pDCP, and tDCP were observed as compared to the control group. Thus, CKD 3 patients had 29% lower absolute numbers of circulating mDCP than controls (*P* < 0.001). Considering pDCP, CKD 3 patients had also significant lower absolute numbers (43% lower) than the control group (*P* < 0.001). Concordantly, absolute tDCP were significantly lower in patients with CKD stage 3 than in controls (*P* < 0.001; Figure 2).

Since relative and absolute numbers of circulating DCP were reduced, the possibility that this decrease was caused by a dilution phenomenon due to an increase of another PBMC could be safely excluded.

### Circulating DCP in patients with CKD stage 3 vs. CAD

For the evaluation of potential coherencies in cardiovascular risk due to DCP changes in patients with proven CAD of different severity [11] and those with CKD stage 3, their levels of circulating DCP were compared (Figure 2).

In patients with CAD, a significant severity-dependent decrease in circulating DCP was observed. Patients with mild CAD had significant higher numbers of circulating mDCP, pDCP, and tDCP than patients with advanced CAD (*P* < 0.001, Figure 2) [11].

In patients with CKD stage 3, absolute numbers of circulating mDCP, pDCP, and tDCP were significantly reduced compared to CAD patients of each score. For CKD 3 vs. advanced CAD, differences were as follows: absolute mDCP (23% lower in CKD 3, *P* = 0.003), pDCP (19% lower, *P* = 0.01) and tDCP (28% lower, *P* < 0.001, Figure 2).

In linear regression analysis, even after adjustment for potential confounders (see method section), the absolute numbers of mDCP, pDCP, and tDCP in the CKD 3 group remained significantly reduced as compared to controls and CAD patients (*P* < 0.001, respectively, see Additional file 1).

### Circulating DCP in CKD 3 patients with CVE vs. CKD 3 patients without CVE

In CKD 3 patients having previous CVE compared to those without CVE, a significant reduction in DCP was observed for the relative numbers of mDCP (0.15% of WBC in CKD 3 with CVE vs. 0.18% of WBC in CKD 3 without CVE, *P* = 0.011) and tDCP (0.23% of WBC in CKD 3 with CVE vs. 0.27% of WBC in CKD 3 without CVE, *P* = 0.003), the absolute numbers of DCP tended to be reduced (Figure 2, for absolute numbers of DCP).

### Estimated GFR and DCP

**Figure 3:**
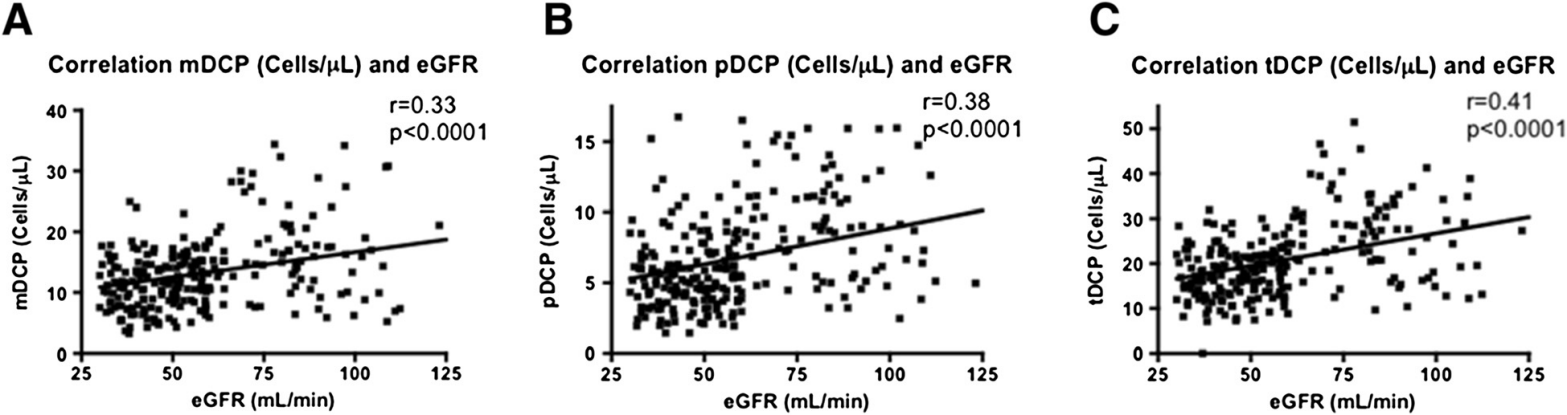
**Correlation of absolute numbers of circulating mDCP, pDCP, tDCP with eGFR in controls and CKD 3 patients without CVE.** Absolute numbers of circulating mDCP **(A)**, pDCP **(B)**, and tDCP **(C)** (cells/μL) of 85 coronary healthy controls and 182 patients with CKD stage 3 without CVE were correlated with the corresponding eGFR (mL/min) (Spearman Rank test).

As illustrated in Figure 3, there was a significant positive correlation between eGFR and circulating DCP in controls and CKD 3 patients without CVE (*P* < 0.0001, respectively).

### Relationship of CRP and DCP

**Figure 4:**
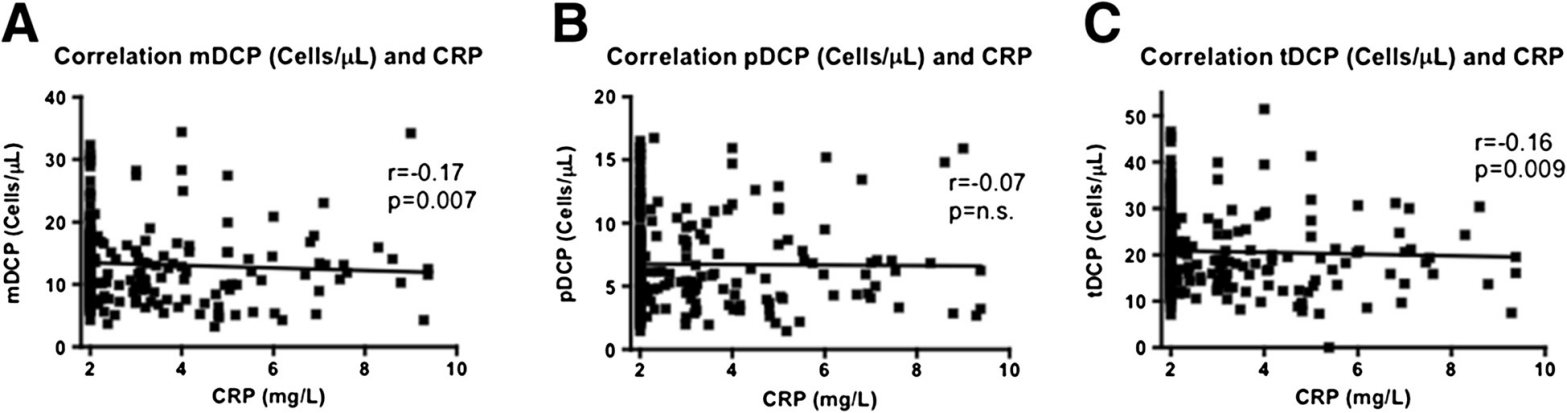
**Correlation of absolute numbers of circulating mDCP, pDCP, tDCP with CRP in controls and CKD 3 patients without CVE.** Absolute numbers of circulating mDCP **(A)**, pDCP **(B)**, and tDCP **(C)** (cells/μL) of 85 coronary healthy controls and 182 patients with CKD stage 3 without CVE were correlated with the corresponding CRP (mg/L) (Spearman Rank test).

According to linear regression analysis, the CRP level had no significant influence on the reduction of DCP in CKD 3 patients as compared to controls and CAD patients (Additional file 1). In patients without any known CAD (controls and CKD 3 patients without CVE), only a weak inverse correlation between CRP and mDCP (r = -0.17, *P* = 0.007) and tDCP (r = -0.16, *P* = 0.009) was found (Figure 4).

## Discussion

CV risk is significantly increased in patients with CKD of any stage. Patients having ESRD are at the highest risk [21]. However, a large number of patients do not reach ESRD because of an earlier death due to CVE. Thus, the CV health status of patients with CKD is of interest for a successful prevention of CVD. Therefore the search for new CV biomarkers is important in CKD patients [13].

In the present study, we investigated the number of circulating DCP in patients with CKD stage 3 compared to coronary healthy controls and patients with stable CAD. A significant decrease of circulating mDCP, pDCP, and tDCP was found in CKD 3 patients compared to controls and patients with known CAD. Although the groups differed in several parameters, linear regression confirmed our findings even if adjusted for possible confounders.

By the current study, a decrease in circulating DCP could be shown in a substantial number of patients with a stage of CKD earlier than ESRD. Previous studies in CKD analysed circulating DCP only in relatively low numbers of patients, predominantly with ESRD. Agrawal *et al*. [2] showed that the pDCP and tDCP count of patients with advanced CKD on dialysis was significantly lower compared to controls. DCP declined further after dialysis, which was explained by the migration to tissues or the accumulation in the dialyzer and tubing system. Lim *et al*. [3] already observed decreased relative DCP numbers in patients with CKD without the need for renal replacement therapy (n = 10, eGFR >50mL/min) and renal transplant recipients.

In CAD patients, however, a significant decrease in circulating DCP compared to controls is well-described [11,22]. In our study, even if compared with patients having advanced CAD, CKD 3 patients had significantly lower numbers of mDCP, pDCP, and tDCP probably reflecting a link to CV risk potentiation in this population. Circulating DCP were significantly reduced in both, CKD 3 without known CAD and advanced CAD without CKD.

In previous studies [11,22], a comparable decrease in circulating DCP was shown for patients with stable and unstable CAD compared with healthy controls (CAD excluded by coronary angiography). In particular, the decrease in circulating mDCP observed by Yilmaz *et al.*[10] revealed another possibility for CV risk prediction as mDCP were significantly reduced even at a stable stage of coronary atherosclerosis. The mDCP are known to be involved in the inflammatory processes related to atherosclerosis. They are present in atherosclerotic plaques [4]. Their number increases in the vascular vessel wall depending on the stage of atherosclerosis, furthermore they promote plaque destabilization [10,11]. In contrast, pDCP counts were only slightly reduced in patients with CAD according to their different migration pattern, function, turnover, kinetics, migration signals, and the different role of both subsets in atherogenesis [10]. However, van Vré *et al.*[22] detected significantly lower relative and absolute numbers of circulating pDCP in patients with CAD, whereas for mDCP a reduction was observed only by trend. The reason for this discrepancy might be that pDCP are specialized in the modulation of immune responses concerning strength, duration, and quality. Depending on the stimulus, pDCP may become either immunostimulating or tolerogenic. Consequently, pDCP have been detected in inflamed peripheral tissues [23]. Van Brussel *et al.*[24] found a significant reduction in both mDCP and pDCP in patients with stable angina pectoris and proven CAD. In contrast to these studies, Shi *et al.*[25] observed an increase in relative and absolute numbers of mDCP in men with proven CAD but pDCP were similar to healthy controls.

A severity-dependent decrease in circulating mDCP, pDCP, and tDCP was shown in patients with CAD underlining the significant association between the decline of circulating DCP and the extent of CAD [11,22]. In the present study, a significant reduction of relative mDCP and tDCP numbers was observed in CKD 3 patients with CVE compared to those without previous CVE. The absolute numbers of mDCP, pDCP, and tDCP in CKD 3 patients with CVE tended also to be reduced compared with those CKD 3 patients without CVE. Moreover, even CKD 3 patients without CVE had significantly reduced absolute numbers of mDCP, pDCP, and tDCP compared with patients having advanced CAD. If DCP are considered to serve as possible CV biomarkers, this may be interpreted as a sign of CV risk equivalency between CKD 3 without CAD and CAD without CKD, at least with respect to DCP counts.

There was a significant positive correlation between the number of DCP and eGFR confirming previous findings [3]*.* The reasons for that are unknown so far. A reversible functional impairment between renal function and DCP generation is discussed [6]. Moreover, a negative impact of the uremic milieu on DC function could be demonstrated [26]. Monocyte-derived DC from patients with advanced CKD showed depressed endocytosis and impaired maturation but increased cytokine production and T-cell proliferation when cultured with uremic sera [26]. The influence of the uremic state on DC generation and function could also be a reason for the decrease in circulating DCP even at CKD stage 3. It can be speculated whether vitamin D deficiency or impaired vitamin D metabolism which both are present in CKD are of importance for this phenomenon. Poor vitamin D status indicates an increased CV risk and could be linked to a compromised innate immune response [27,28]. Thus, Takeda *et al.* demonstrated that oral calcitriol administration led to a marked reduction in atherosclerotic lesion formation in a mouse model. This reduction was explained by the suppression of immune reactions, especially by changing the function or differentiation of DC and regulatory T-cells [29]. Reduced production of DCP in the bone marrow is also possible but patients with CKD stage 5 showed no deficiencies in serum levels of granulocyte macrophage colony-stimulating factor (GM-CSF), a DC mobilizing cytokine [3]. Levels of leukocyte subpopulations other than DC were not reduced in the blood of our CKD cohort (data not shown). Whether increased apoptosis of circulating DCP due to CKD contributes to reduced DCP levels needs to be established. Another reason for a decrease in DCP could be an increased recruitment and elevated turnover of circulating DCP in chronically inflamed tissues all over the body including the vessel wall. Several studies about DCP reduction in patients with atherosclerosis and CVD describe a stage-dependent accumulation of mDC and pDC in vascular lesions [11,22]. Recruitment of mDC from blood into the intima is induced by several proatherogenic factors which also suppress the recirculation of mDC from the vessel wall into the blood [11,22]. Moreover, recent studies from our group detected significantly elevated levels of mDC in the infarcted area of patients with myocardial infarction compared to healthy myocardium highlighting enhanced recruitment and consumption of DCP into the target tissue as well as elevated recruitment of inflammatory cells like macrophages and T cells [9].

Furthermore there was a weak but significant inverse correlation between CRP and mDCP and tDCP. *Van Vré et al.*[30] showed that an increased CRP level leads to the activation of mDC and an increased expression of DC maturation markers in vitro. Moreover a decreased migration of mDC was observed in response to increasing concentrations of CRP [30]. The number of pDC was unaffected [30,31] as it was the case for pDCP in our study.

Although linear regression did not show a significant influence of age on our findings, there was a weak influence of age on DCP in the control group. Concerning this, it is known that aging is characterized by a decline in immune functions and by manifold changes in the micro-environment that could affect activation and/or maturation of DC [32,33]. Accordingly, Hawiger *et al.*[34] observed an increased age-associated circulation of proinflammatory mediators that can trigger the activation and maturation of DC.

## Conclusion

Dendritic cell precursors are significantly decreased in patients with CKD stage 3 as compared to coronary healthy subjects and patients with CAD. Circulating DCP might be of relevance for CV risk potentiation in patients with CKD. Whether circulating DCP in CKD may act as new CV biomarkers needs to be established in prospective endpoint studies. Since DCP correlate positively with eGFR, the causes for DCP reduction in CKD need to be investigated.

### Limitations

Whether CKD 3 patients without CVE might have suffered from clinically silent CAD cannot be excluded since a coronary angiography was not undertaken in this previously cardiovascular healthy cohort. Furthermore, CAD-patients were recruited in the University hospital of Erlangen during a different period of time than the CKD 3 patients.

## Competing interests

The authors declare that they have no competing or financial competing interests.

## Authors’ contributions

The authors’ contributions were as follows: KP, DK, AY and MB suggested, drafted and promoted this paper. KP, DK, AY, ST, GW and MB were responsible for patient recruitment and FACS analyses. BB was responsible for clinical data processing. KP and DK performed statistical analyses and DK was responsible for figure preparation. KP, DK, AY, GW, MB took action in interpretation of data. AY, ST, GW and MB revised the manuscript critically. All authors read and approved the final version of the manuscript.

## Pre-publication history

The pre-publication history for this paper can be accessed here:

http://www.biomedcentral.com/1471-2369/14/274/prepub

## Supplementary Material

Additional file 1 **Linear regression analysis - results.** A linear regression was performed using DCP count as dependent variables. Age, male gender, diabetes mellitus, hypertension, smoking, CRP, leukocyte count, GFR, creatinine, cholesterol, HDL, LDL and TG and group dependence were analysed as independent variables. For group dependency, CKD patients were used as the reference group. As a result the absolute mDCP, pDCP, and tDCP numbers in the CKD 3 group remained significant reduced compared to controls and CAD patients even if adjusted for the confounders (*P* < 0.001, respectively). Click here for file

## The GCKD Study Investigators

Regional Study Centres

Technical University of Aachen, Germany

Frank Eitner, MD

Georg Schlieper, MD

Katharina Findeisen, MD MPH

Elfriede Arweiler, MPH

Sabine Ernst, MSc

Mario Unger, RN

Jürgen Floege, MD

Charité, Humboldt-University of Berlin, Germany

Elke Schaeffner, MD, MSc

Seema Baid-Agrawal, MD

Kerstin Petzold, RN

Ralf Schindler, MD

University of Erlangen-Nürnberg, Germany

Karl F. Hilgers, MD

Silvia Hübner, MD

Susanne Avendano, RN

Dinah Becker-Grosspietsch, RN

University of Freiburg, Germany

Anna Köttgen, MD, MPH

Ulla Schultheiss, MD

Simone Meder, RN

Erna Mitsch, RN

Gerd Walz, MD

Hannover Medical School, Germany

Johan Lorenzen, MD

Jan T. Kielstein, MD

Petra Otto, RN

Hermann Haller, MD

University of Heidelberg, Germany

Claudia Sommerer, MD

Claudia Föllinger, RN

Tanja Löschner, RN

Martin Zeier, MD

University of Jena, Germany

Martin Busch, MD

Katharina Paul, MSc

Lisett Dittrich

Gunter Wolf, MD, MHBA

Ludwig-Maximilians University of München, Germany

Thomas Sitter, MD

Robert Hilge, MD

Claudia Blank

University of Würzburg, Germany

Vera Krane, MD

Daniel Schmiedeke, MD

Sebastian Toncar, MD

Daniela Cavitt, RN

Christoph Wanner, MD

Stefan Franz, MD

Study and data coordinating center:

University of Erlangen-Nürnberg, Germany

Kai-Uwe Eckardt, MD (PI)

Stephanie Titze, MD

Nina Hauck, MSc

Susanne A. Seuchter, BSc

Birgit Hausknecht

Marion Rittmeier

Anke Weigel

Hans-Ulrich Prokosch, PhD

Barbara Bärthlein, BSc

Kerstin Haberländer, BSc

Andreas Beck, MSc

Thomas Ganslandt, MD

Stefanie Stefan, MSc

Sabine Knispel, PhD

Thomas Dressel, MSc

Olaf Gefeller, PhD

Matthias Schmid, PhD

Martina Malzer, BSc

Analytical centres:

University of Erlangen-Nürnberg, Germany

Institute of Human Genetics

André Reis, MD

Arif B. Ekici, PhD

Innsbruck Medical University, Germany

Division of Genetic Epidemiology

Florian Kronenberg, MD

Barbara Kollerits, PhD

Hansi Weißensteiner, MSc

Lukas Forer, MSc

Sebastian Schönherr

University of Regensburg, Germany

Institute of Functional Genomics

Peter Oefner, PhD

Wolfram Gronwald, PhD
