# Supplementary material for: Circulating dendritic cell precursors in chronic kidney disease: a cross-sectional study
Source: BMC Nephrol. 2013 Dec 10;14:274. doi: 10.1186/1471-2369-14-274 (PMC3878881; doi:10.1186/1471-2369-14-274)
Supplement: Additional file 1 — Linear regression analysis - results. A linear regression was performed using DCP count as dependent variables. Age, male gender, diabetes mellitus, hypertension, smoking, CRP, leukocyte count, GFR, creatinine, cholesterol, HDL, LDL and TG and group dependence were analysed as independent variables. For group dependency, CKD patients were used as the reference group. As a result the absolute mDCP, pDCP, and tDCP numbers in the CKD 3 group remained significant reduced compared to controls and CAD patients even if adjusted for the confounders (P < 0.001, respectively). [file 1471-2369-14-274-S1.doc]

**Additional files**

**Table 1 Linear regression analysis - results**

| **Dependend variable** |  | **Coefficient (SE)** | ***P*-Value** |
| --- | --- | --- | --- |
| mDCP abs.  (cells per µL) | Age [years] | 0,034 | 0,083 |
|  | Gender (0=female, 1=male) | 0,705 | 0,830 |
|  | Diabetes mellitus (0=no, 1=yes) | 0,712 | 0,155 |
|  | Hypertension (0=no, 1=yes) | 0,903 | 0,050 |
|  | Smoking (0=no, 1=yes) | 0,645 | 0,431 |
|  | CRP [mg/L] | 0,166 | 0,409 |
|  | Leukocyte count [Gpt/L] | 0,181 | <0,001 |
|  | GFR [mL/min/1.73m^2^] | 0,028 | 0,336 |
|  | Creatinine [mg/dL] | 1,115 | 0,847 |
|  | Cholesterol [mg/dL] | 0,032 | 0,414 |
|  | HDL [mg/dL] | 0,037 | 0,508 |
|  | LDL[mg/dL] | 0,034 | 0,261 |
|  | TG [mg/dL] | 0,005 | 0,492 |
|  | Controls vs CKD 3 patients | 1,257 | <0,001 |
|  | CAD patients vs CKD 3 patients | 1,201 | <0,001 |
|  | Intercept | 4,450 | 0,008 |
|  |  |  |  |
| pDCP abs.  (cells per µL) | Age [years] | 0,021 | 0,040 |
|  | Gender (0=female, 1=male) | 0,428 | 0,814 |
|  | Diabetes mellitus (0=no, 1=yes) | 0,433 | 0,214 |
|  | Hypertension (0=no, 1=yes) | 0,549 | 0,314 |
|  | Smoking (0=no, 1=yes) | 0,392 | 0,793 |
|  | CRP [mg/L] | 0,101 | 0,570 |
|  | Leukocyte count [Gpt/L] | 0,110 | <0,001 |
|  | GFR [mL/min/1.73m^2^] | 0,017 | 0,111 |
|  | Creatinine [mg/dL] | 0,677 | 0,766 |
|  | Cholesterol [mg/dL] | 0,019 | 0,278 |
|  | HDL [mg/dL] | 0,022 | 0,404 |
|  | LDL[mg/dL] | 0,021 | 0,256 |
|  | TG [mg/dL] | 0,003 | 0,274 |
|  | Controls vs CKD 3 patients | 0,764 | <0,001 |
|  | CAD patients vs CKD 3 patients | 0,730 | <0,001 |
|  | Intercept | 2,704 | 0,013 |
|  |  |  |  |
| tDCP abs.  (cells per µL) | Age [years] | 0,047 | 0,005 |
|  | Gender (0=female, 1=male) | 0,958 | 0,877 |
|  | Diabetes mellitus (0=no, 1=yes) | 0,968 | 0,581 |
|  | Hypertension (0=no, 1=yes) | 1,229 | 0,041 |
|  | Smoking (0=no, 1=yes) | 0,877 | 0,684 |
|  | CRP [mg/L] | 0,226 | 0,887 |
|  | Leukocyte count [Gpt/L] | 0,247 | <0,001 |
|  | GFR [mL/min/1.73m^2^] | 0,038 | 0,113 |
|  | Creatinine [mg/dL] | 1,516 | 0,838 |
|  | Cholesterol [mg/dL] | 0,043 | 0,349 |
|  | HDL [mg/dL] | 0,050 | 0,451 |
|  | LDL[mg/dL] | 0,046 | 0,249 |
|  | TG [mg/dL] | 0,007 | 0,422 |
|  | Controls vs CKD 3 patients | 1,709 | <0,001 |
|  | CAD patients vs CKD 3 patients | 1,633 | <0,001 |
|  | Intercept | 6,051 | 0,001 |
|  |  |  |  |
